# Complete AV Block From Septal Cardiac Metastases of Anal Squamous Carcinoma Managed With Leadless Pacing

**DOI:** 10.1016/j.jaccas.2026.110522

**Published:** 2026-09-26

**Authors:** Emiliano Venditti, Silvia Ialungo, Camilla Cocchi, Serena Bracci, Luca Tagliaferri, Emanuele Marzetti, Francesco Landi, Giuseppe Ferdinando Colloca

**Affiliations:** a Department of Geriatrics, Orthopedics and Rheumatology, Università Cattolica del Sacro Cuore, Rome, Italy; b Fondazione Policlinico Universitario “Agostino Gemelli” IRCCS, Rome, Italy; c Radiation Oncology, Department of Diagnostic Imaging and Radiation Oncology, Fondazione Policlinico Universitario Agostino Gemelli IRCCS, Rome, Italy; d Department of Geriatrics, Orthopaedics and Rheumatological Sciences, Fondazione Policlinico Universitario Agostino Gemelli IRCCS, Rome, Italy; e Department of Imaging Diagnostic, Oncologic Radiotherapy and Haematology, Fondazione Policlinico Universitario Agostino Gemelli IRCCS, Rome, Italy

**Keywords:** anal squamous cell carcinoma, atrioventricular block, cardiac metastases, cardio-oncology, leadless pacemaker

## Abstract

**Background:**

Cardiac metastases from anal squamous cell carcinoma (ASCC) are exceedingly rare; complete atrioventricular (AV) block from septal involvement has not previously been reported In Vivo.

**Case Summary:**

A 79-year-old woman with p16-negative ASCC developed complete AV block with asystolic pauses during chemoradiotherapy. Restaging computed tomography showed a 49-mm interventricular septal mass and 2 left ventricular lesions, consistent with cardiac metastases. A Micra AV leadless pacemaker was implanted because of pacemaker dependence, frailty, infection risk, and challenging vascular access. Cemiplimab, an anti–PD-1 immunotherapy, was initiated for progressive metastatic disease.

**Discussion:**

Septal metastatic infiltration was the most likely cause of the AV block. New conduction disease in patients with cancer warrants multimodality imaging to assess cardiac involvement.

**Take-Home Messages:**

Leadless pacing can be a practical option for complete AV block in frail oncology patients. Cardiac metastases should be considered in new arrhythmias or conduction disturbances, particularly with septal lesions.

## Visual Summary

**Figure:**
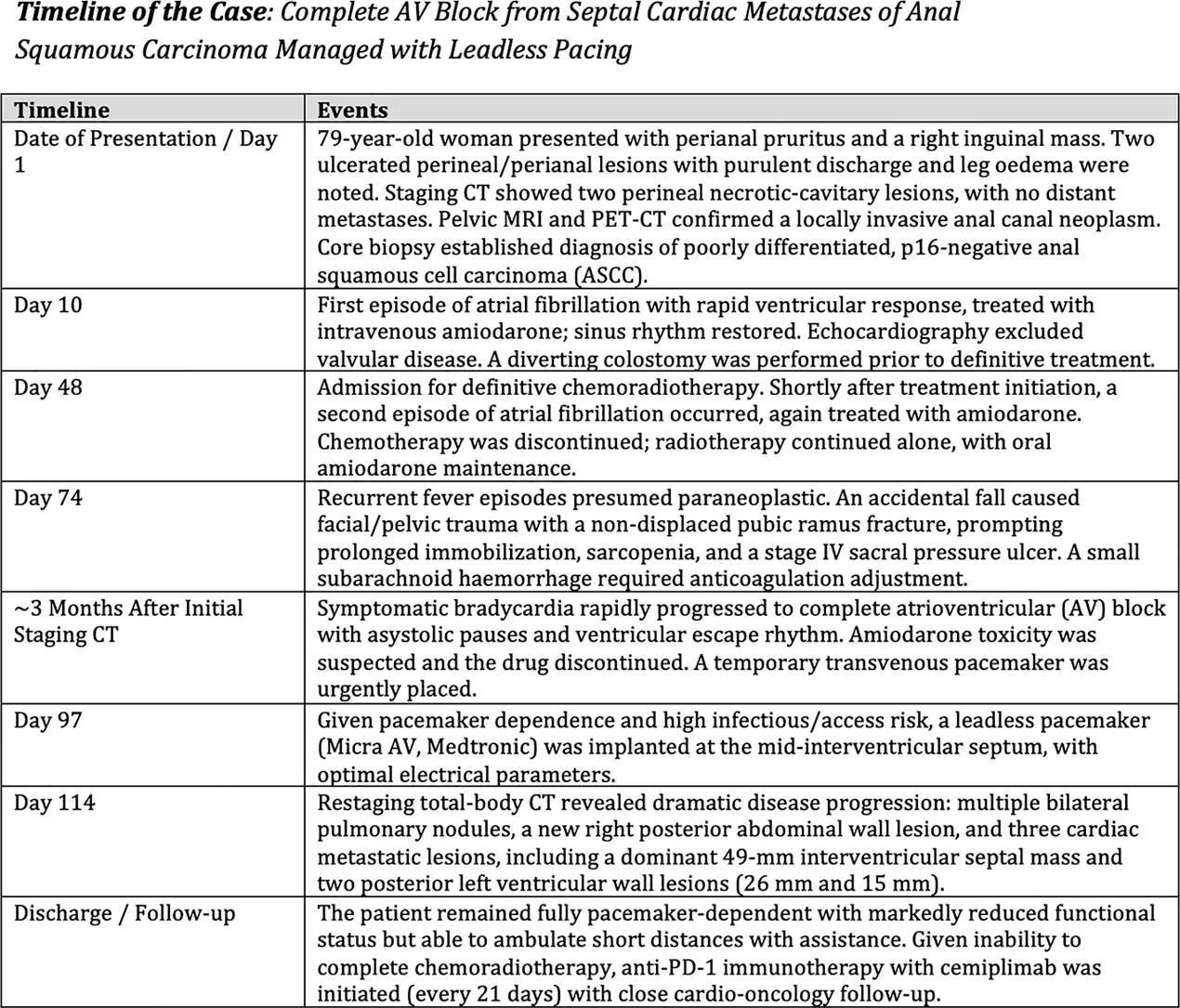

## History of Presentation

A 79-year-old woman presented with intense perianal pruritus and a right inguinal mass. Two ulcerated perineal/perianal lesions with purulent discharge and left greater than right leg edema were noted, with progressive worsening over the following months.

Total-body computed tomography (CT) documented 2 solid perineal lesions with irregular margins and necrotic-cavitary features (Figure 1): an anterior lesion between the right labium majus and the ipsilateral adductor compartment (48 × 33 mm, craniocaudal extension 37 mm), and a posterior perianal lesion extending into the right ischioanal fossa (23 × 31 mm). Bilateral necrotic inguinal lymphadenopathy was present, more pronounced on the right (up to 13 mm short axis). No cerebral, pulmonary, or other distant lesions were identified.

**Figure 1:**
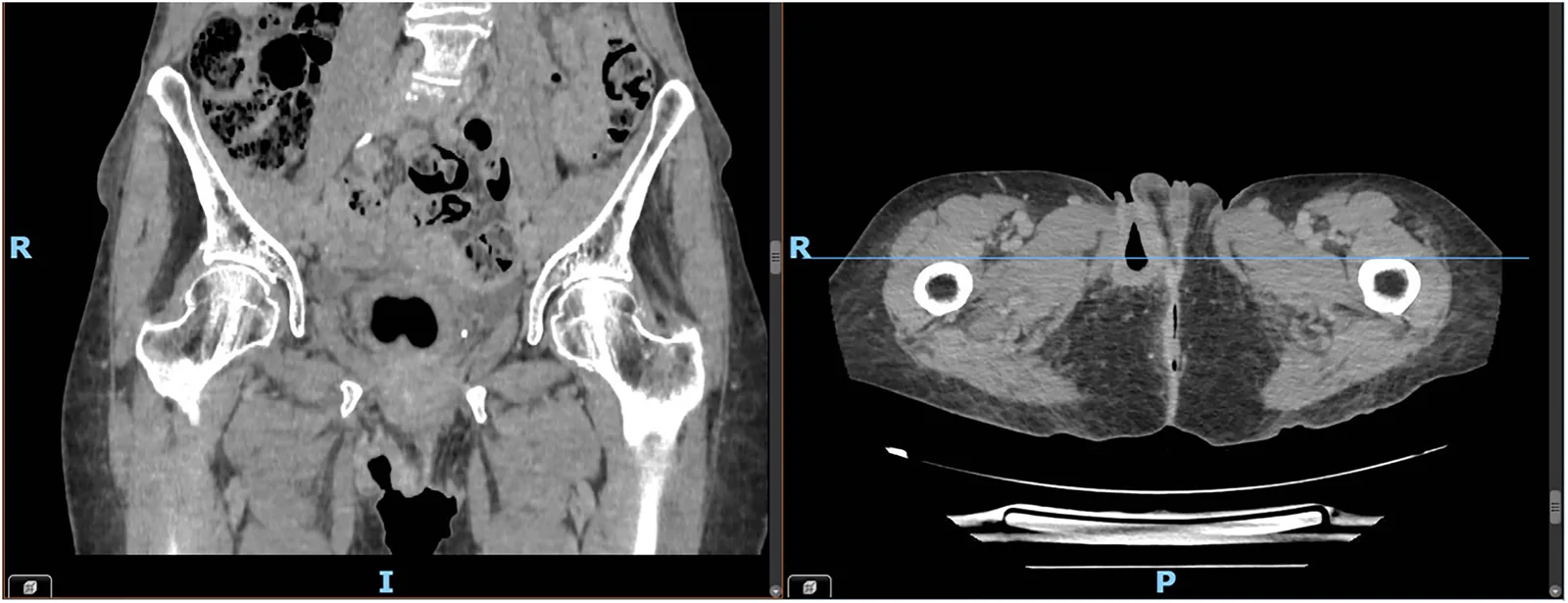
Initial Radiologic Investigations Contrast-enhanced computed tomography of the perineal region showing 2 cavitated lesions with heterogeneous enhancement.

Pelvic magnetic resonance imaging confirmed an anal canal neoplasm with 33-mm craniocaudal extension, infiltrating the right internal and external sphincters and extending to the ipsilateral ischioanal fossa (Figure 2). Positron emission tomography-CT showed intense metabolic activity (SUV_max_ 28.1 posterior lesion; 23.5 anterior lesion), confirming high biological aggressiveness.

**Figure 2:**
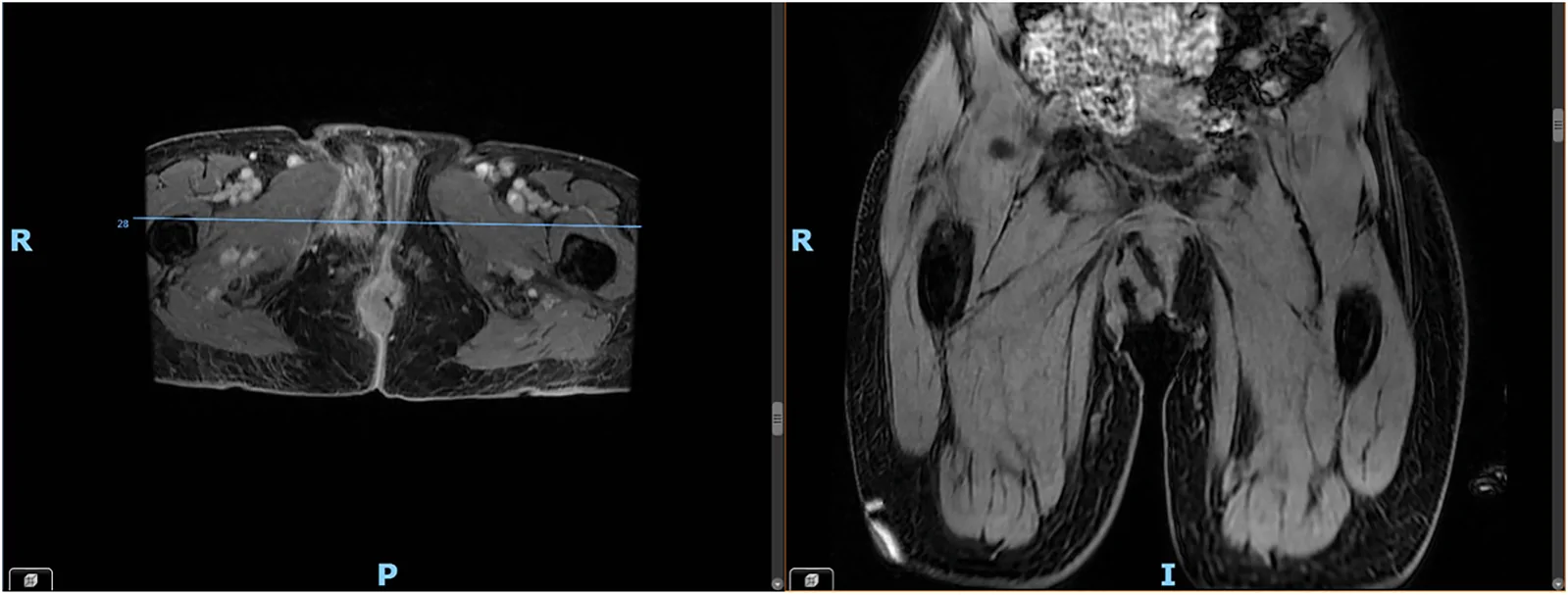
Pelvic Magnetic Resonance Imaging Anal canal lesion infiltrating internal and external sphincters with extension to the ischioanal fossa and anterior fistulization.

Core needle biopsy of the inguinal ulcerated lesion demonstrated a malignant epithelioid neoplasm positive for AE1/AE3, CAM5.2, p63, and p40, and negative for p16, consistent with poorly differentiated squamous carcinoma.

During the initial hospitalization, the patient developed a first episode of atrial fibrillation with rapid ventricular response, treated with intravenous amiodarone with restoration of sinus rhythm. Echocardiography excluded a valvular etiology, showing normal biventricular function without significant valvulopathy or pericardial effusion.

Given the local extent of disease, a diverting laparoscopic colostomy was performed without complications before definitive chemoradiotherapy.

## Management

Approximately 1 month later, the patient was readmitted to initiate definitive treatment with pelvic radiotherapy and concomitant fluorouracil. Three days after initiation, a second episode of atrial fibrillation occurred, again requiring amiodarone. Chemotherapy was discontinued, and radiotherapy continued alone with oral amiodarone maintenance.

The hospital course was complicated by recurrent fever, presumed paraneoplastic, and by an accidental fall with facial and pelvic trauma. Brain and facial CT were negative for acute injury, but pelvic radiography revealed a nondisplaced right pubic ramus fracture, requiring nonweight-bearing status. Prolonged immobilization led to sarcopenia and a stage IV sacral pressure ulcer; anticoagulation was adjusted after a small subarachnoid hemorrhage on brain magnetic resonance imaging.

Approximately 3 months after the initial staging CT, the patient developed symptomatic bradycardia rapidly progressing to complete atrioventricular (AV) block with asystolic pauses and ventricular escape rhythm (Figure 3). Amiodarone toxicity was suspected, and the drug was discontinued. A temporary transvenous pacemaker was urgently placed. Given pacemaker dependence, infection risk, and difficult iliofemoral access, a leadless pacemaker (Micra AV; Medtronic) was implanted at the mid-interventricular septum, achieving optimal electrical parameters (sensing 12.5 mV, impedance 610 Ω, threshold 0.5 V).

**Figure 3:**
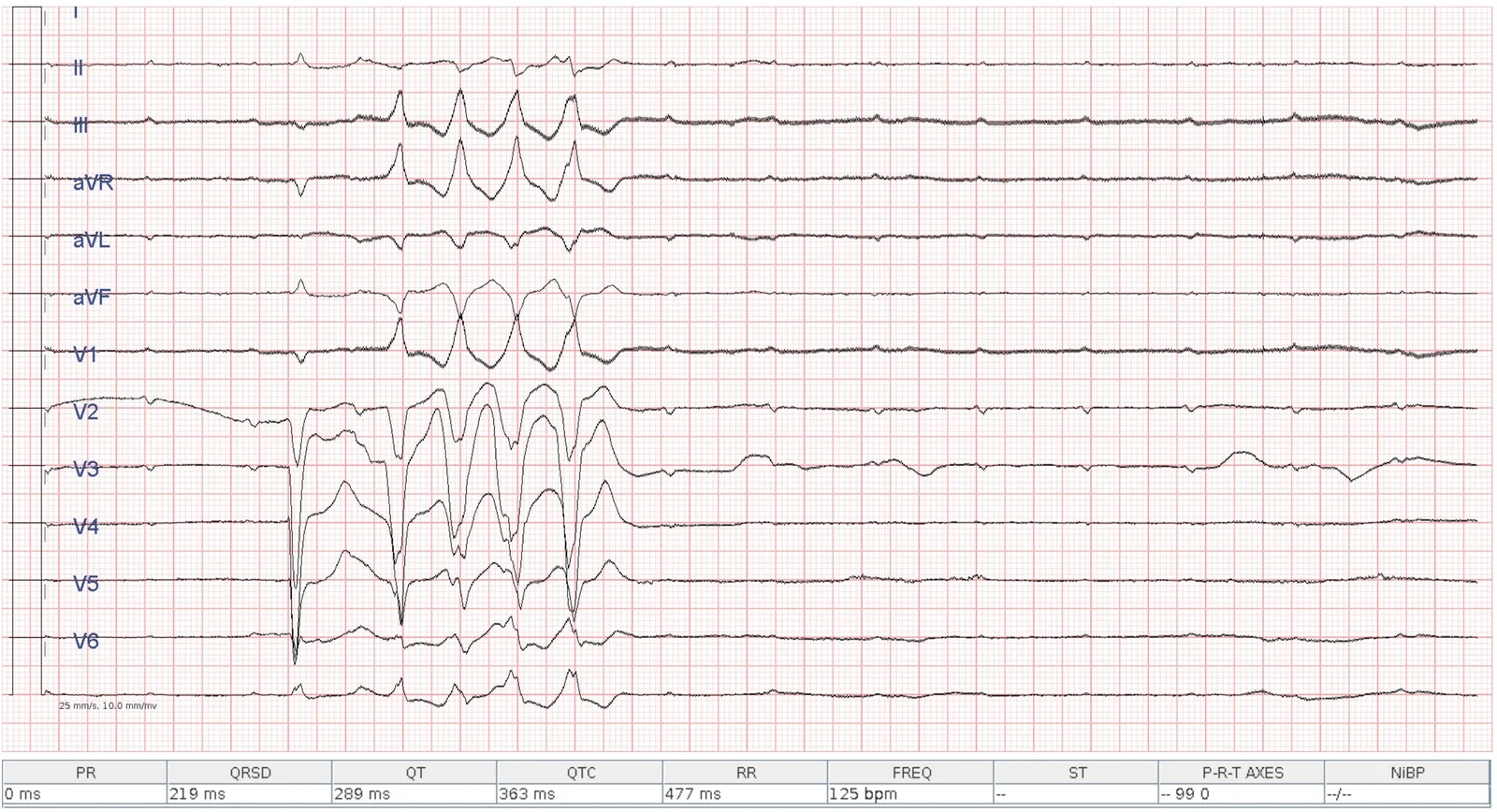
Complete Atrioventricular Block With Ventricular Escape Rhythm and Nonsustained Ventricular Tachycardia 12-lead electrocardiogram showing nonconducted P waves consistent with complete atrioventricular block, prolonged pauses/asystolic episodes, and a wide-QRS complex ventricular escape rhythm; a brief run of nonsustained ventricular tachycardia is also evident.

Approximately 3 weeks later, total-body restaging CT was performed before further radiotherapy (Figure 4) and revealed dramatic progression: multiple bilateral pulmonary nodules (at least 7, largest 18 mm), a new right posterior abdominal wall lesion infiltrating the quadratus lumborum muscle, and, most notably, 3 cardiac lesions with soft-tissue attenuation consistent with metastases—a dominant 49-mm interventricular septal mass and 2 posterior left ventricular wall lesions (26 and 15 mm). Perianal disease remained stable, whereas inguinal lymphadenopathy progressed.

**Figure 4:**
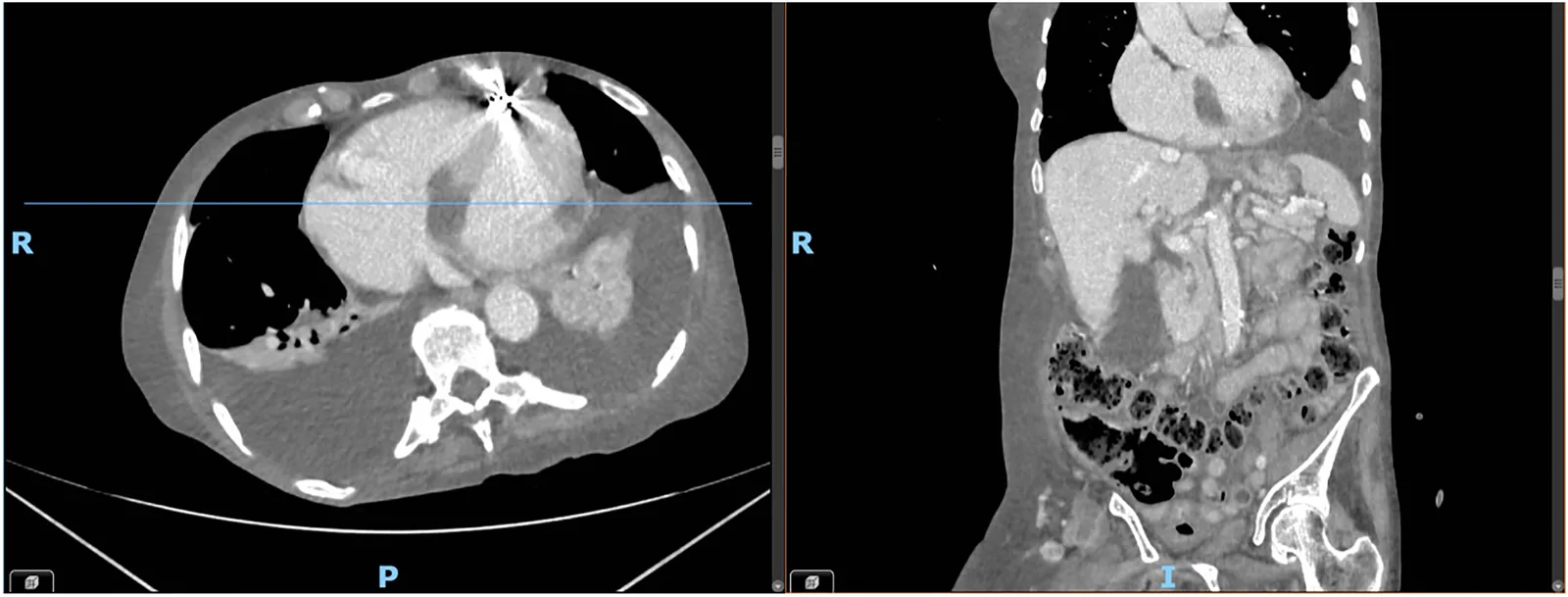
Restaging Contrast-Enhanced Computed Tomography Three cardiac masses, including a dominant 49-mm interventricular septal mass and 2 posterior left ventricular wall lesions.

The patient was fully pacemaker-dependent, with severely compromised activities of daily living, though able to stand and ambulate short distances with assistance. Given systemic progression and inability to complete chemoradiotherapy, anti-programmed death-1 (PD-1) immunotherapy with cemiplimab (every 21 days) was initiated, an off-label choice supported by class efficacy of PD-1 inhibitors in pretreated squamous carcinoma.

## Discussion

Anal squamous cell carcinoma (ASCC) is rare, with an annual incidence of 10.7-15.5 cases per million.^1^ Most patients present with locoregional disease; distant dissemination occurs in 10%-20% of cases, typically involving extra-pelvic lymph nodes, liver, and lung.^2^

Cardiac metastases, though more common than primary cardiac tumors (approximately 40:1), remain rare in clinical practice.^3^ Autopsy series report cardiac involvement in 2.3%-18.3% of oncologic patients (average 7.1%), but most lesions are clinically silent, explaining the marked discrepancy with the in vivo diagnostic rate of <1%.^4^

Tumors with the greatest propensity for cardiac spread include melanoma, mesothelioma, lung, breast, and esophageal carcinoma, and lymphoma, reflecting anatomic proximity and vascularization. Squamous cell carcinomas overall account for cardiac metastases in only 5%-10% of autopsy series, and head-and-neck squamous carcinoma is an especially rare cause, with fewer than a dozen in vivo cases reported.^5^

Isolated cardiac metastases have been described from squamous carcinomas of the tongue, larynx, and esophagus, but cardiac involvement from ASCC appears even more exceptional. Our literature search identified no prior in vivo case of cardiac metastasis from ASCC causing complete AV block, underscoring the novelty of this report. Most reports of atypical ASCC spread describe mediastinal, duodenal, or cerebral localization; cardiac involvement is exceptionally rare. In our patient, metastases specifically involved the ventricular myocardium, with a dominant septal mass and 2 left ventricular wall lesions, consistent with the myocardium being the most frequently involved cardiac site after the pericardium.^6^

The arrhythmic presentation, culminating in complete AV block, was initially attributed to amiodarone toxicity; however, massive infiltration of the interventricular septum—the site of the conduction system—likely contributed to or directly caused the conduction disturbance, a recognized complication of septal metastases that can manifest as atrial arrhythmias, bundle branch block, or AV block of varying degree.^7^

Leadless pacemakers avoid subcutaneous pockets and transvenous leads, reducing device-related infection and lead/pocket complications, at the cost of a small but higher periprocedural perforation risk. In frail oncology patients with contaminated fields or active infection, concomitant extraction and immediate leadless reimplantation have shown favorable outcomes with reduced length of stay. Systemic therapy in pretreated metastatic ASCC increasingly relies on PD-1 blockade; nivolumab and pembrolizumab have shown durable responses in subsets of patients. Although cemiplimab is not specifically approved for ASCC, it was selected here based on logistical and tolerability considerations and strong evidence of activity in advanced cutaneous squamous cell carcinoma.^8^

## Follow-Up

At discharge, the patient remained pacemaker-dependent with markedly reduced functional status but able to transfer and ambulate short distances with assistance. Cemiplimab was scheduled every 21 days, with close cardio-oncology follow-up.

## Conclusions

In older patients with oncology, new conduction disease or arrhythmias should prompt consideration of cardiac metastases, particularly with septal involvement. Leadless pacing offers a pragmatic strategy to minimize infectious and access-related complications in frail patients, and anti-PD-1 therapy remains a rational option in pretreated metastatic ASCC, individualized to clinical context.

**Take-Home Messages**

• In a patient with cancer, rapidly evolving bradyarrhythmia or complete atrioventricular block should prompt a search for reversible causes and for structural cardiac disease, including myocardial metastatic involvement of the interventricular septum.
• When permanent pacing is required in a frail patient with active malignancy, high infection risk, or limited venous access, a leadless pacemaker may offer a feasible alternative to a conventional transvenous system.

## Funding Support and Author Disclosures

The authors have reported that they have no relationships relevant to the contents of this paper to disclose.

**Visual Summary:** Timeline of the Case

Timeline	Events
Date of presentation/Day 1	79-y-old woman presented with perianal pruritus and a right inguinal mass. Two ulcerated perineal/perianal lesions with purulent discharge and leg edema were noted. Staging CT showed 2 perineal necrotic-cavitary lesions, with no distant metastases. Pelvic MRI and PET-CT confirmed a locally invasive anal canal neoplasm. Core biopsy established diagnosis of poorly differentiated, p16-negative anal squamous cell carcinoma (ASCC).
Day 10	First episode of atrial fibrillation with rapid ventricular response, treated with intravenous amiodarone; sinus rhythm restored. Echocardiography excluded valvular disease. A diverting colostomy was performed before definitive treatment.
Day 48	Admission for definitive chemoradiotherapy. Shortly after treatment initiation, a second episode of atrial fibrillation occurred, again treated with amiodarone. Chemotherapy was discontinued; radiotherapy continued alone, with oral amiodarone maintenance.
Day 74	Recurrent fever episodes presumed paraneoplastic. An accidental fall caused facial/pelvic trauma with a nondisplaced pubic ramus fracture, prompting prolonged immobilization, sarcopenia, and a stage IV sacral pressure ulcer. A small subarachnoid hemorrhage required anticoagulation adjustment.
∼3 Mo after initial staging CT	Symptomatic bradycardia rapidly progressed to complete atrioventricular (AV) block with asystolic pauses and ventricular escape rhythm. Amiodarone toxicity was suspected, and the drug was discontinued. A temporary transvenous pacemaker was urgently placed.
Day 97	Given pacemaker dependence and high infectious/access risk, a leadless pacemaker (Micra AV; Medtronic) was implanted at the mid-interventricular septum, with optimal electrical parameters.
Day 114	Restaging total-body CT revealed dramatic disease progression: multiple bilateral pulmonary nodules, a new right posterior abdominal wall lesion, and 3 cardiac metastatic lesions, including a dominant 49-mm interventricular septal mass and 2 posterior left ventricular wall lesions (26 mm and 15 mm).
Discharge/follow-up	The patient remained fully pacemaker-dependent with markedly reduced functional status but able to ambulate short distances with assistance. Given inability to complete chemoradiotherapy, anti-PD-1 immunotherapy with cemiplimab was initiated (every 21 d) with close cardio-oncology follow-up.

Timeline summarizing the clinical course of an elderly woman with anal squamous cell carcinoma who developed complete atrioventricular block because of septal cardiac metastases, successfully managed with a leadless pacemaker, followed by initiation of anti-PD-1 immunotherapy.

AV = atrioventricular; CT = computed tomography; MRI = magnetic resonance imaging; PET-CT = positron emission tomography-computed tomography; PD-1 = programmed death-1.
